# HPV-independent vulvar squamous cell carcinoma: a case report and review of the literature

**DOI:** 10.31744/einstein_journal/2025RC1482

**Published:** 2025-10-13

**Authors:** Raphael Federicci Haddad, Mari Hattori Ballantyne Wyper, Samuel Drumond Esperança, Gustavo Yano Callado, Karla Calaça Kabbach Prigenzi, Fernanda Kesselring Tso, Patricia Napoli Belfort-Mattos

**Affiliations:** 1 Hospital Israelita Albert Einstein São Paulo SP Brazil Hospital Israelita Albert Einstein, São Paulo, SP, Brazil.; 2 Hospital Municipal Dr. Moysés Deutsch Hospital Israelita Albert Einstein São Paulo SP Brazil Hospital Municipal Dr. Moysés Deutsch; Hospital Israelita Albert Einstein, São Paulo, SP, Brazil.; 3 Hospital Israelita Albert Einstein Faculdade Israelita de Ciências da Saúde Albert Einstein São Paulo SP Brazil Faculdade Israelita de Ciências da Saúde Albert Einstein, Hospital Israelita Albert Einstein, São Paulo, SP, Brazil.

**Keywords:** Vulvar neoplasms, Squamous cell carcinoma, Oncologic surgery, Papillomavirus infections, Female genital neoplasms

## Abstract

Vulvar cancer, a rare gynecological malignancy, accounts for 3-5% of cases and is most commonly squamous cell carcinoma. Prognosis is poorer in cancers unrelated to human papillomavirus (HPV), often associated with chronic inflammation. We report the case of a 42-year-old woman from São Paulo, Brazil, who presented with a vulvar lesion initially misdiagnosed as an ingrown hair. Despite treatment, the lesion progressed, and subsequent evaluation revealed invasive squamous cell carcinoma. Biopsy confirmed HPV-independent disease, with immunohistochemistry demonstrating aberrant p53 expression. Molecular analysis identified differentiated vulvar intraepithelial neoplasia, underscoring the distinct molecular pathways of HPV-related and HPV-independent carcinogenesis. This case emphasizes the importance of early diagnosis, comprehensive histopathological assessment, and further research to improve management and outcomes in HPV-independent vulvar cancer.

## INTRODUCTION

Vulvar cancer is a rare malignancy, representing 3-5% of gynecological neoplasms.^(1)^ The majority of cases are squamous cell carcinoma (SCC; ∼75%), while less common histological subtypes include melanoma, basal cell carcinoma, Bartholin's gland adenocarcinoma, sarcoma, and Paget's disease.^(1)^

The incidence of vulvar cancer peaks in the seventh and eighth decades of life and has shown an overall increase in recent decades.^(2)^ Contributing factors include rising rates of smoking, alcohol consumption, unsafe sexual practices, and human immunodeficiency virus (HIV) infection. While the incidence of human papillomavirus (HPV)-associated vulvar cancers has increased, non-HPV-related cancers have remained relatively stable.^(3)^

Vulvar carcinogenesis follows two distinct pathways: HPV-dependent and HPV-independent. The HPV-dependent pathway, more frequent in younger women, is associated with usual-type vulvar intraepithelial neoplasia and may involve *PIK3CA* activating mutations.^(4)^ By contrast, the HPV-independent pathway is linked to chronic inflammatory conditions such as lichen sclerosus and involves differentiated vulvar intraepithelial neoplasia (dVIN) as a precursor lesion.^(4)^ Although the mechanisms underlying this pathway are not fully understood, dVIN is associated with an elevated risk of progression to invasive SCC, often with a shorter latency compared to usual vulvar intraepithelial neoplasia.

The *TP53* mutation is a key driver of non-HPV-related vulvar SCC, with immunohistochemistry (IHC) frequently demonstrating aberrant p53 expression.^(5)^ However, a subset of HPV-negative tumors display wild-type *TP53* expression and are characterized by an intermediate prognosis, with a five-year survival rate of ∼64%.^(6)^ These tumors may harbor alterations in *CDKN2A* and components of the NOTCH signaling pathway, suggesting a distinct, truly HPV-independent origin.^(6)^

Among the risk factors for HPV-independent vulvar SCC, lichen simplex chronicus (LSC) is notable. LSC develops from chronic physical trauma associated with pruritic lesions and is most often observed in patients in their fourth to sixth decades of life. Its diagnosis is primarily based on characteristic histopathological features.^(7)^

Clinically, HPV-independent vulvar SCCs generally have worse outcomes than their HPV-associated counterparts. They are associated with reduced overall, disease-specific, and recurrence-free survival, and carry a higher risk of lymph node metastasis.^(8)^ Lymph node involvement remains the most important independent prognostic factor for both survival and local recurrence, even in cases with negative surgical margins.^(8)^

The molecular heterogeneity and distinct clinical behavior of HPV-independent vulvar SCC underscore the need to consider vulvar cancer as a diverse disease entity. This case report of non-HPV-related vulvar SCC highlights the importance of understanding its unique pathophysiology to refine treatment strategies and improve patient outcomes.

## CASE REPORT

We report the case of a 42-year-old woman of African descent from São Paulo, Brazil, referred by primary care to a specialized lower genital tract pathology service for evaluation of a vulvar lesion.

The patient initially noted the lesion, which she presumed to be an "ingrown hair," and attempted treatment with manual expression and antibiotic therapy, without improvement. The lesion progressively enlarged, prompting specialized medical evaluation approximately 90 days after onset.

Her medical history included grade I obesity, poorly controlled systemic arterial hypertension due to medication non-adherence, and a 15 pack-year smoking history. Serologies for syphilis and HIV were negative. Gynecological history revealed a vaginal delivery 11 years earlier and a recent oncotic cytology negative for intraepithelial lesions or malignancy. She reported no additional symptoms or comorbidities.

At the initial consultation, the patient presented results of an incisional biopsy performed in primary care. The biopsy (0.5cm fragment) showed a papillary lesion with cytoarchitectural alterations suggestive of viral involvement but without evidence of malignancy.

**Figure 1:**
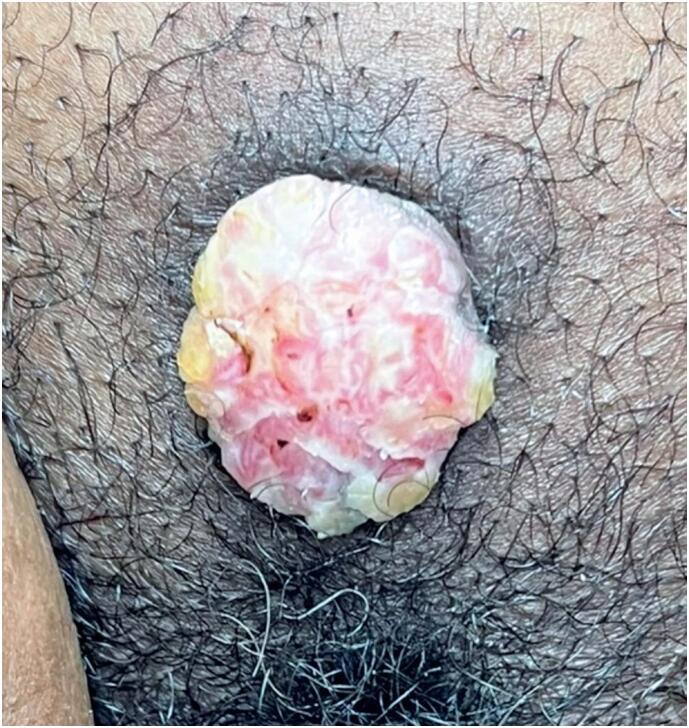
Lesion of the pubic mound

On physical examination, a tumor located on the pubic mound along the midline was identified, measuring 25×20mm. The lesion was warty, whitish, firm-elastic, and mildly tender to palpation, with slight bleeding upon friction but no inguinal lymphadenopathy or signs of infection (Figure 1). Surgical excision with 1cm margins was performed under local anesthesia on September 12, 2022.

**Figure 2:**
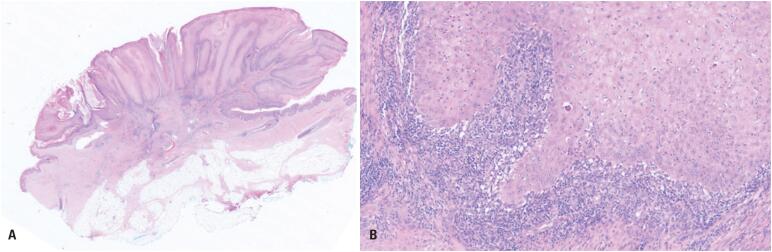
Invasive squamous cell carcinoma of the vulva, HPV-independent, with a predominantly exophytic architecture (10× magnification) (A) and detail of infiltrative growth (100× magnification) (B)

**Figure 3:**
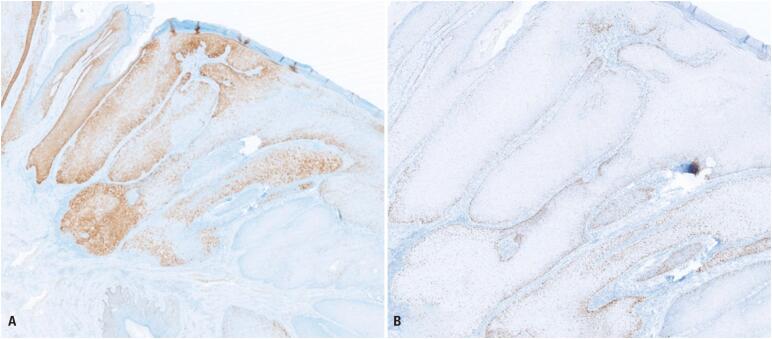
Immunohistochemistry: (A) normal mosaic expression of p16 protein (40× magnification), excluding HPV-related etiology; (B) abnormal p53 expression with overexpression (100× magnification), supporting HPV-independent pathogenesis

Histopathological analysis revealed invasive SCC, HPV-independent, associated with dVIN, with a depth of invasion of 0.8mm (Figure 2). The nearest peripheral margin was 8mm from the invasive front, with no angiolymphatic invasion. Immunohistochemistry demonstrated normal mosaic expression of p16 and abnormal p53 expression with an overexpression pattern (Figure 3). Hybridization testing for high-risk (HPV-16, −18, −31, −33, −35, −39, −45, −51, −52, −56, −58, −66) and low-risk (HPV-6, −11) HPV DNA was negative. Pathological staging was pT1b according to the AJCC (2017), corresponding to stage IB in the FIGO (2018) system. Following diagnosis, the patient was referred to a specialized oncology service for further management.

Written informed consent was obtained for the use of de-identified clinical data and medical images. The consent process included detailed explanation of study objectives, potential risks and benefits, and patient rights, with ample opportunity for questions prior to signature.

The study was approved by the Institutional Research Ethics Committee of the *Secretaria Municipal da Saúde de São Paulo - SMS/SP* (CAAE: 70548223.5.0000.0086; approval #6.632.300).

## DISCUSSION

HPV-associated SCC generally carries a more favorable prognosis than HPV-independent SCC.^(8)^ Current literature emphasizes the clinical significance of determining HPV status, as it influences both prognosis and surgical management.^(8)^ There is a trend toward less invasive surgery for HPV-related vulvar SCC, while HPV-independent cases often require more aggressive surgical approaches. Given the marked genetic and clinical differences between these two pathways, further clarification is needed regarding the role of adjuvant therapies, including targeted therapy and radiation, and whether treatment strategies should differ accordingly.^(8)^

HPV-associated SCC is more frequently observed in younger women, often presents as multifocal disease, is linked to classic vulvar intraepithelial neoplasia, and may co-occur with squamous neoplasms of the lower genital tract.^(9)^

Approximately 10% of vulvar neoplasms are associated with dVIN, although this percentage is likely underestimated due to underdiagnosis. In this case, dVIN was identified alongside invasive SCC, consistent with previous reports. Early recognition of dVIN is crucial, as timely diagnosis and appropriate management of precursor lesions represent key strategies for preventing progression to invasive carcinoma.^(2)^ Histopathological and molecular analyses are essential for distinguishing HPV-related from HPV-independent vulvar cancer, with significant implications for treatment and prognosis.^(6)^ In the present case, an initial incisional biopsy performed in primary care reported only morphological features and yielded discordant findings compared with the excisional biopsy. This discrepancy may reflect the absence of molecular analysis, underscoring the importance of integrating molecular biology into diagnostic workflows.

Immunohistochemistry showed normal p16 expression and aberrant p53 expression (overexpression pattern), findings consistent with HPV-independent vulvar SCC. These align with evidence that *TP53* mutations and alterations in the NOTCH signaling pathway are characteristic of HPV-independent carcinogenesis.^(4,10)^ In a study by Kortekaas et al., 413 cases of vulvar SCC were stratified into three subtypes based on HPV and p53 status: HPV-negative/p53-mutated, HPV-negative/p53-wild type, and HPV-positive. The HPV-negative/p53-mutated group, which accounted for 66% of cases and corresponds to the present report, demonstrated the poorest overall survival, relative survival, and recurrence-free survival.^(9)^ The authors proposed that this classification could guide treatment and follow-up strategies.

This case illustrates the diagnostic complexity of HPV-independent vulvar SCC and highlights the importance of recognizing associated inflammatory lesions not traditionally emphasized, such as lichen sclerosus or lichen simplex chronicus. Personalized diagnostic and therapeutic approaches are needed, with particular attention to pathogenic mechanisms involving *TP53* mutations and associated risk factors, to improve prevention, diagnosis, and treatment strategies in this rare malignancy.

Microscopic features of dVIN include abnormal keratinocyte maturation and basal layer atypia, characterized by hyperchromasia, nuclear enlargement, irregular nuclear contours, and prominent nucleoli. Regarding invasive SCC, depth of invasion remains the most important prognostic factor in stage I disease.^(8)^ In this case, the reported depth of invasion was 0.8mm, measured from the base of the lesion to the most superficial adjacent dermal papilla, as recommended by the 2018 FIGO criteria. An alternative method, measuring from the basal layer of dysplastic epithelium, has been proposed and correlates with clinical outcome and prognosis, but it remains under validation and was included in the FIGO 2021 review. Using this method, invasion depth in the present case was 0.2mm.

Finally, differentiating dVIN from conditions such as lichen simplex chronicus, lichen sclerosus, and pseudoepitheliomatous hyperplasia can be challenging. In this context, *TP53* mutation status serves as a valuable diagnostic marker to support accurate distinction.

## CONCLUSION

Vulvar squamous cell carcinoma is a complex malignancy that requires comprehensive evaluation for effective management. Histopathological and immunohistochemical analyses are essential for accurate diagnosis and staging, while molecular techniques play a critical role in characterizing atypical vulvar lesions. Integrating these diagnostic approaches with individualized therapeutic strategies offers the potential to improve patient outcomes and quality of life.
